# Decoupling in a joint communication and sensing system with metasurface

**DOI:** 10.1038/s41598-026-44469-6

**Published:** 2026-03-22

**Authors:** Zizhen Zhang, Zirui Zhang, Ziqi Ren, Zhirun Hu

**Affiliations:** Department of Electrical and Electronic Engineering, University of Manchester, Manchester, UK

**Keywords:** Joint communication and sensing, Multiple-input multiple-output (MIMO), Metasurface, Modified split-ring resonator, Mutual coupling suppression, Self-interference (SI) cancellation, Engineering, Physics

## Abstract

The increasing demand for integrated communication and sensing has led to the development of Joint Communication and Sensing (JCAS) systems. However, strong self-interference (SI) between transmitting (TX) and receiving (RX) antennas remains a major obstacle, significantly degrading system performance in compact MIMO arrays. Traditional signal-processing-based cancellation methods face limitations in wideband scenarios due to high complexity and potential signal distortion. In this work, a novel metasurface-assisted decoupling structure is presented. The metasurface based on modified split-ring resonators (MSRRs) can suppress surface currents and reduce the coupling between TX and RX arrays. To further enhance isolation and reduce front-end self-interference in sensing-centric full-duplex JCAS, a multi-frequency null-space projection (NSP) beamforming algorithm is integrated with the antenna array design, forming a hardware–algorithm co-optimization framework. As proof of concept, a 2 $\times$ 2 patch antenna array incorporating the proposed metasurface operating in the 9–10 GHz band has been designed, fabricated, and characterized. The measurement results validate effectiveness. The findings suggest that the proposed decoupling approach offers a promising solution for enhancing electromagnetic isolation and overall system performance in next-generation JCAS applications such as intelligent transportation and indoor wireless sensing.

## Introduction

In recent years, Multiple Input Multiple Output (MIMO) technology has gained widespread applications in wireless systems for its ability to significantly enhance data throughput and signal robustness. As smart devices proliferate and the Internet of Things (IoT) expands, traditional communication systems face increasing limitations in meeting the dual demands of high-speed data transmission and accurate environmental sensing^1–3^. This has spurred the development of Joint Communication and Sensing (JCAS) systems (also known as Integrated Sensing and Communication (ISAC), which aim to unify wireless communication and sensing functionalities in a single platform to support emerging applications such as autonomous driving and intelligent infrastructure^4–6^.

A critical challenge in JCAS systems lies in the strong self-interference (SI) due to coupling between transmitting (TX) and receiving (RX) antennas^7^. Unlike conventional systems that merely suppress interference, JCAS architectures must simultaneously preserve useful reflected signals for sensing while minimizing leakage from the TX path^8^. Recent advances in weighted beamforming methods, particularly those introducing frequency nulls, have shown promise in mitigating SI^9^. However, these methods often suffer from increased system complexity and potential signal distortion at specific frequencies, especially when deployed across wideband systems^10^.

In the context of JCAS system design, recent research has explored both algorithmic and structural approaches for SI mitigation. Beamforming-based signal processing methods can effectively suppress interference in the spatial domain yet often require precise channel state information (CSI) and complex matrix operations, particularly when applied over wide bandwidth^11^. Meanwhile, decoupling structures such as electromagnetic bandgap (EBG)^12^, defected ground structures (DGS)^13^, and mushroom metasurfaces^14^ have been studied to reduce mutual coupling between antenna elements. However, many of these designs are either limited in bandwidth, large in size, or difficult to integrate into compact planar antenna arrays^15–17^. The modified split-ring resonator (MSRR) proposed in this work offers a compact alternative, capable of achieving strong surface current suppression within a targeted frequency band. Furthermore, recent studies have highlighted that mutual coupling not only degrades communication quality through signal leakage and reduced isolation, but also adversely affects the sensing performance by distorting reflected signal characteristics^18^. This dual degradation underscores the necessity of a joint hardware- and algorithm-design optimization approach. The design of compact, high-isolation antenna arrays using metamaterials such as MSRRs thus holds strategic value for enabling efficient JCAS deployments in complex electromagnetic environments.

Recent studies have demonstrated metasurface/metamaterial-enabled functionality integration beyond conventional applications. For example, intelligent programmable metasurface systems combined with computer vision and neural-network-assisted control have been reported to realize automatic tracking of moving targets and wireless communications^19^. Moreover, externally perceivable smart leaky-wave antennas based on spoof surface plasmon polaritons (SSPPs) have also been proposed to support adaptive radiating/non-radiating switching and frequency-controlled beam tracking with computer-vision assistance^20^. In parallel, stretchable antenna arrays for conformal deployment on curved platforms have been developed to maintain stable electromagnetic performance under bending/deformation^21^. These advances highlight a clear trend toward intelligent and conformal integrated EM platforms; in this context, compact front-end SI suppression for co-located TX/RX full-duplex JCAS remains a key enabling functionality that is addressed in this work.

To address these limitations, this work proposes a hardware-based SI suppression strategy through the integration of metamaterial-inspired decoupling structures. Specifically, an MSRR metasurface is designed to suppress surface currents and reduce inter-array coupling. When excited by incident electromagnetic waves, the resonant behavior of MSRRs induces localized magnetic response, which redistributes surface current and improves isolation. While some recent JCAS works, especially some commercial radar/sensing platforms (especially automotive radars/sensors) adopt millimeter-wave bands to exploit larger bandwidth and compact apertures, the sensing function is not restricted to millimeter-wave operation and can also be implemented at microwave bands such as X-band. In this work, we focus on sensing-centric front-end SI suppression (antenna-domain coupling reduction and coupling-matrix-based beamforming), rather than on maximizing range resolution via extremely wide bandwidth. Therefore, the 9–10 GHz band is chosen as a proof-of-concept platform for reliable fabrication and measurement. The proposed MSRR metasurface is resonant and band-selective, but the concept can be adapted to other bands (including mmWave) through geometry re-optimization and tolerance-aware implementation on appropriate low-loss substrates. This work is focused on the RF/antenna-domain SI coupling channel between co-located TX/RX arrays, which is a waveform-agnostic bottleneck in full-duplex JCAS. Therefore, beyond isolation/coupling reduction, we quantify the communication-relevant impact using the SINR metric in Eq. (7) and the corresponding results in Fig. 9, rather than presenting an end-to-end baseband communication decoding demonstration. The rest of this paper is organized as follows: Section II introduces the beamforming and decoupling algorithm design. Section III presents the structure and electromagnetic behavior of the MSRR-based metasurface. Section IV discusses simulation and measurement results. Finally, Section V concludes the work and outlines future research directions.

## Algorithm design

The JCAS system studied in this work consists of two arrays, namely the transmitting (TX) and the receiving (RX) arrays, which respectively contain Nt transmitting antennas and Nr receiving antennas^22^. The transmission and reception operations are carried out simultaneously in the same frequency band, forming a full duplex communication architecture^23^. The transmitting beam simultaneously carries the functions of the communication beam and the radar beam, and adopts the dynamic weight allocation strategy^9^. The transmit waveform at the TX can be expressed as,

1 $$\begin{aligned} x(t) = {\textbf{w}}_{\textrm{TX}} \, s(t) \end{aligned}$$

where s(t) is a general frequency-domain TX symbol. The TX beam consists of both the communication and the radar beams, so the weights of the TX beam can be denoted as^9^,

2 $$\begin{aligned} {\textbf{w}}_{\textrm{TX}} = \sqrt{\rho } \, {\textbf{w}}_{\textrm{TX,C}} + \sqrt{1 - \rho } \, {\textbf{w}}_{\textrm{TX,R}} \end{aligned}$$

where $\rho$ denotes the power distribution between the communication and radar beams, with $0 \le \rho \le 1$. The weights of the communication ${\textbf{w}}_{\textrm{TX},C}$ and radar sensing ${\textbf{w}}_{\textrm{TX},R}$ beams are calculated respectively through the following direction vectors,

3 $$\begin{aligned} {\textbf{w}}_{\textrm{TX,C}} = \frac{{\textbf{a}}_{\textrm{TX}}^*(\theta _{\textrm{C}})}{\left\| {\textbf{a}}_{\textrm{TX}}(\theta _{\textrm{C}}) \right\| }, \quad {\textbf{w}}_{\textrm{TX,R}} = \frac{{\textbf{a}}_{\textrm{TX}}^*(\theta _{\textrm{R}})}{\left\| {\textbf{a}}_{\textrm{TX}}(\theta _{\textrm{R}}) \right\| } \end{aligned}$$

The TX communication and radar/sensing array responses are represented by $a_{\textrm{TX}}(\theta _{C})$ and $a_{\textrm{TX}}(\theta _{R})$. while $\theta _{C}$ and $\theta _{R}$ denote the angle of communication and the angle of radar, respectively.

The JCAS front-end consists of an $N_t$-element transmitting (TX) array and an $N_r$-element receiving (RX) array. A narrowband normalized signal model is adopted at each frequency sample, where the beamforming weights are applied in post-processing to the measured multi-port coupling channel.

To support joint communication and radar/sensing, ${\textbf{w}}_{\textrm{TX}}$ is formed by a communication beam and a radar/sensing beam:

4 $$\begin{aligned} {\textbf{w}}_{\textrm{TX}}(\theta _{\textrm{R}})=\sqrt{\rho }\,{\textbf{w}}_{\textrm{TX,C}}(\theta _{\textrm{C}}) +\sqrt{1-\rho }\,{\textbf{w}}_{\textrm{TX,R}}(\theta _{\textrm{R}}),\qquad 0\le \rho \le 1, \end{aligned}$$

where $\theta _{\textrm{C}}$ and $\theta _{\textrm{R}}$ denote the communication and radar steering angles, respectively.

Let ${\textbf{H}}_{\textrm{SI}}(f_n)\in {\mathbb {C}}^{N_r\times N_t}$ denote the frequency-selective TX–RX SI coupling coupling matrix at the *n*-th sampled frequency $f_n$, extracted from the measured multi-port S-parameters. Specifically, ${\textbf{H}}_{\textrm{SI}}(f_n)$ is assembled from the measured multi-port S-parameters by collecting the complex transmission coefficients from each TX port to each RX port at frequency $f_n$ (with all other ports terminated by matched 50-$\Omega$ loads). Therefore, ${\textbf{H}}_{\textrm{SI}}(f)$ characterizes the frequency-selective passive TX–RX leakage channel of the antenna/metasurface front-end. In active full-duplex operation, the dominant front-end SI component is the transmitted waveform filtered by this passive leakage channel (under the linear operation assumption of the passive front-end). As a result, applying the beamforming/combining weights to the measured ${\textbf{H}}_{\textrm{SI}}(f_n)$ yields the effective residual SI at the RX combiner output, whose complex gain is given by $g_{\textrm{SI}}(f_n)$ in (5). The corresponding residual SI power is proportional to $|g_{\textrm{SI}}(f_n)|^2$, and the band-averaged residual SI power gain is quantified by $C_{\textrm{ave}}$ in (6). Hence, the average residual SI power term can be expressed as $P_{\textrm{TX}}\,C_{\textrm{ave}}$, which is used in the normalized SINR in (7). It should be noticed that the SINR in (7) is normalized to indicate the relative SINR change caused by SI reduction (with $P_{\textrm{sig}}$ normalized to unity and $P_n$ set to a fixed value), rather than an end-to-end link-level performance with modulated waveforms and radar/sensing target-echo measurements. For a given RX combining vector ${\textbf{w}}_{\textrm{RX}}\in {\mathbb {C}}^{N_r\times 1}$, the beamformed residual SI at $f_n$ becomes the scalar^9^

5 $$\begin{aligned} g_{\textrm{SI}}(f_n)={\textbf{w}}_{\textrm{RX}}^{\textrm{H}}\,{\textbf{H}}_{\textrm{SI}}(f_n)\,{\textbf{w}}_{\textrm{TX}}(\theta _{\textrm{R}}). \end{aligned}$$

Accordingly, the path-averaged residual SI coupling (power gain) over *N* frequency samples is^9^

6 $$\begin{aligned} C_{\textrm{ave}}(\theta _{\textrm{R}})=\frac{1}{N}\sum _{n=1}^{N}\left| g_{\textrm{SI}}(f_n)\right| ^2 =\frac{1}{N}\sum _{n=1}^{N}\left| {\textbf{w}}_{\textrm{RX}}^{\textrm{H}}\,{\textbf{H}}_{\textrm{SI}}(f_n)\,{\textbf{w}}_{\textrm{TX}}(\theta _{\textrm{R}})\right| ^2. \end{aligned}$$

Since this work focuses on the communication-relevant impact of SI mitigation (rather than a full end-to-end link-budget measurement), we report a normalized SINR at the output of the RX combiner, where the desired-signal power is set to a constant $P_{\textrm{sig}}$ (normalized to unity in this work), while the SI power scales with the TX power and $C_{\textrm{ave}}$:

7 $$\begin{aligned} \textrm{SINR}(\theta _{\textrm{R}})=\frac{P_{\textrm{sig}}}{P_{\textrm{TX}}\;C_{\textrm{ave}}(\theta _{\textrm{R}})+P_n}, \end{aligned}$$

and $\textrm{SINR}_{\textrm{dB}}(\theta _{\textrm{R}})=10\log _{10}\!\big (\textrm{SINR}(\theta _{\textrm{R}})\big )$, where $P_{\textrm{TX}}$ is the transmit power, and $P_n$ is the receiver noise power.

In this work, hardware–algorithm co-optimization refers to a system-level co-design and joint evaluation of (i) the MSRR metasurface that reduces the TX-to-RX coupling channel, and (ii) the multi-frequency NSP beamforming/combining that further suppresses the effective residual SI under the JCAS beam constraints. The implementation workflow is as follows:

1. Hardware design: tune the MSRR unit-cell dimensions and the $2\times 3$ superstrate layout in full-wave simulations to reduce the dominant TX–RX coupling over 9–10 GHz while maintaining antenna matching.
2. Channel characterization: obtain the frequency-selective SI coupling matrix ${\textbf{H}}_{\textrm{SI}}(f_n)$ from simulated/measured multi-port S-parameters by extracting the TX-to-RX submatrix (TX ports: 1 and 3; RX ports: 2 and 4).
3. Beam definition: for a given communication direction $\theta _{\textrm{C}}$ and radar/sensing scan angle $\theta _{\textrm{R}}$, compute the steering vectors and form ${\textbf{w}}_{\textrm{TX}}=\sqrt{\rho }\,{\textbf{w}}_{\textrm{TX,C}}+\sqrt{1-\rho }\,{\textbf{w}}_{\textrm{TX,R}}$.
4. NSP processing and evaluation: compute the NSP-based weights (applied in post-processing) using ${\textbf{H}}_{\textrm{SI}}(f_n)$, and evaluate the effective residual SI via $g_{\textrm{SI}}(f_n)$, $C_{\textrm{ave}}$, and the normalized SINR using Eqs. (5)–(7).

## Metasurface design and optimization

The MSRR is a metamaterial element structure similar to the split-ring resonator (SRR)^25–27^, When an incident field impinges on the ring structure, the magnetic field component normal to the ring plane induces circulating currents along the metal path, while intense electric fields concentrate across the gaps. The interaction between the circulating current and the strong gap fields leads to a pronounced resonant response at a well defined frequency^25^. Near this resonance, the amplitude and phase of the surface current on the metallic traces are substantially reconfigured, creating regions of current suppression and field enhancement. This reconfiguration modifies the local surface impedance and the scattered field, weakens surface-wave propagation on the substrate, and reduces the dominant near-field coupling paths between neighboring elements. As a result, electromagnetic energy transfer across the structure can be controlled within the target band, which supports lower mutual coupling and improved isolation. The resonance frequency and the strength of the response are governed by the geometry and materials of the MSRR. Gap width controls the electric-field concentration and the rate of energy exchange across the slit. The azimuthal position of the gap determines the phase progression of the current around the ring and therefore the directivity and polarization sensitivity of the scattered field. Ring size and trace width set the effective current path length and current density, which shift the resonance and alter the quality of the response^28–30^. For multi-ring MSRRs, the spacing between rings adjusts the near-field coupling, which provides additional knobs for tuning the resonance and broadening the usable band. The substrate permittivity and thickness further influence the field distribution in and around the cell, which affects the resonance frequency, the usable bandwidth, and the dissipative loss. Compared with a single-gap SRR, the multi-gap and multi-ring variants offer more design degrees of freedom, enabling finer control of current steering, angular response, and polarization behavior within a similar footprint. Practical implementation requires careful control of the relative placement and orientation of the MSRR superstrate and the antenna metallization, tight tolerances on gap and spacing, and high-quality lamination to avoid air voids that shift the resonance and reduce notch depth. It is also necessary to verify that the added structure does not unduly degrade input matching or radiation efficiency. With rational design of the MSRR and array layout, the metasurface therefore enables reliable control of electromagnetic propagation in the desired band and delivers measurable improvements in isolation and overall system performance.

**Table 1:** Parameter of MSRR.

Parameter	Value (mm)	Parameter	Value (mm)
L1	30.0	G3	0.6
L2	17.4	G4	3.7
L3	14.2	G5	0.6
L4	4.0	R1	6.0
L5	5.0	R2	3.8
L6	2.0	W1	90
G1	1.0	W2	29.5
G2	1.0	W3	15

**Fig. 1:**
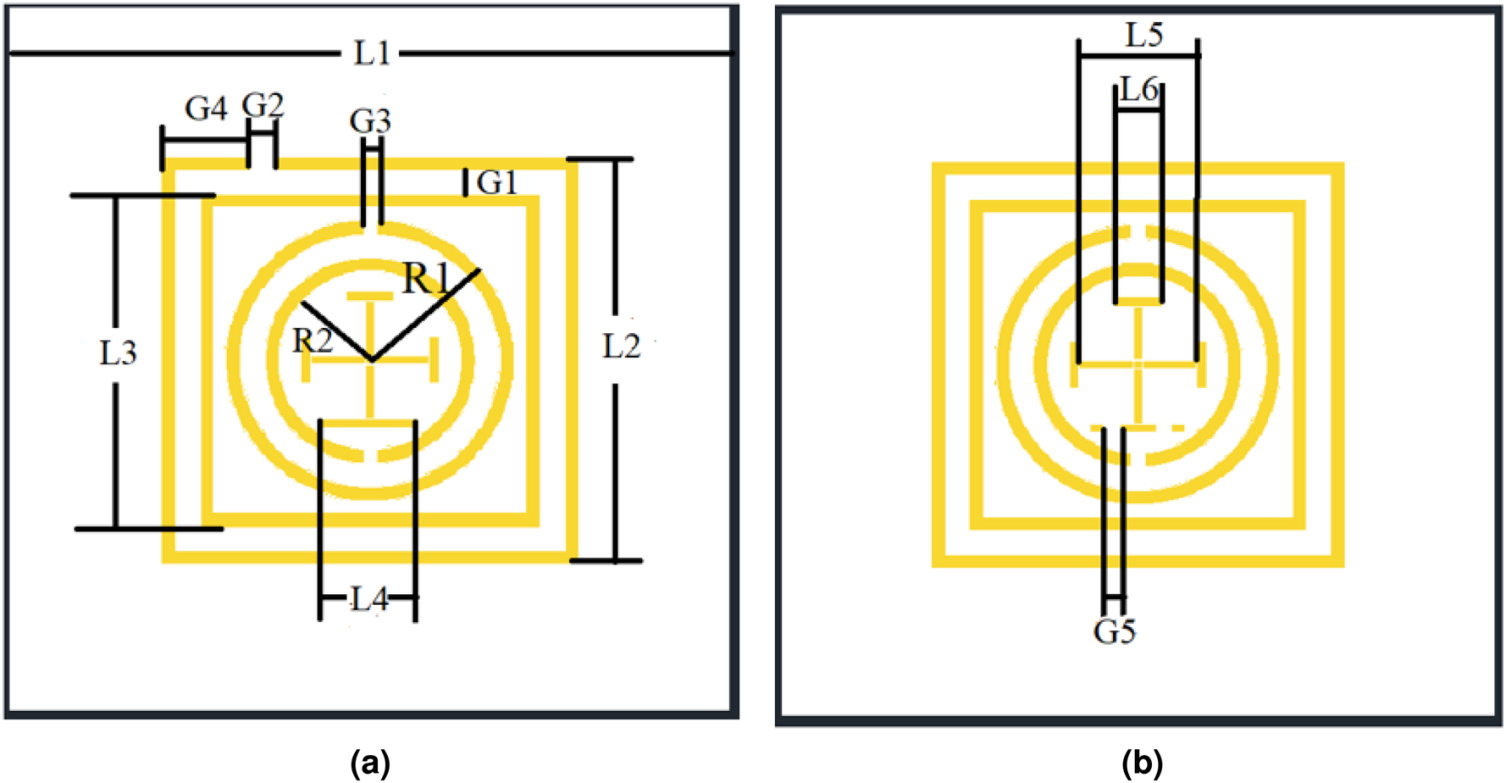
Elemental cells of the proposed MSRR design: (**a**) MSRR1; (**b**) MSRR2.

**Fig. 2:**
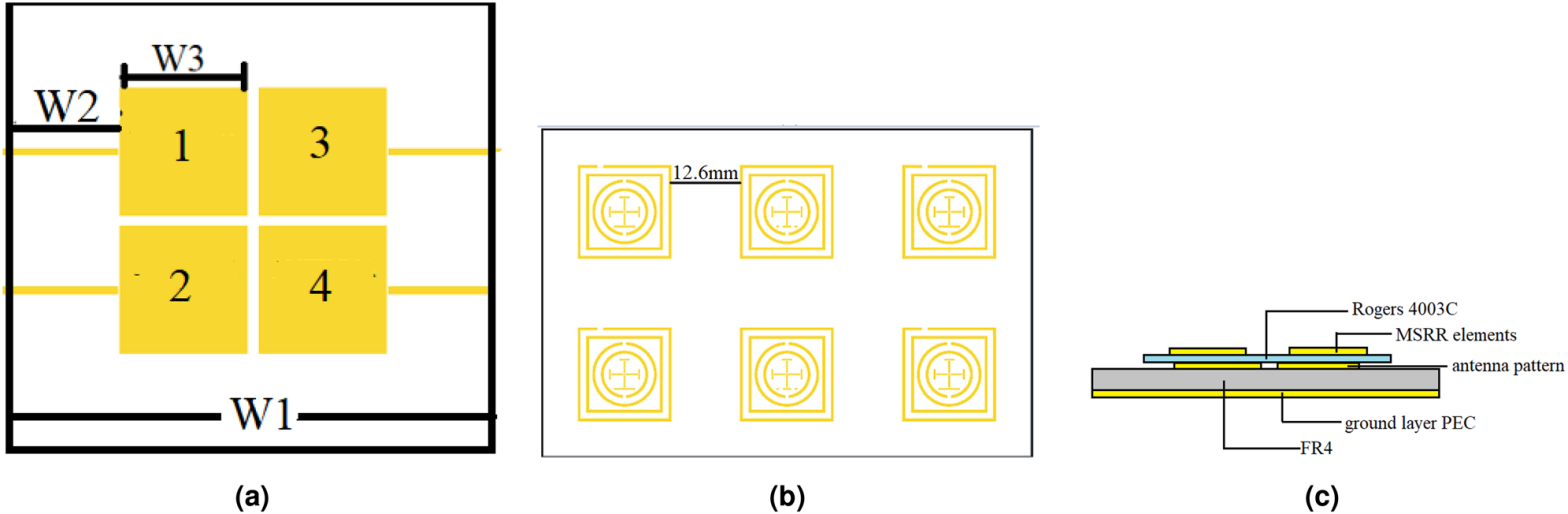
(**a**) MIMO antenna array, (**b**) MSRR surface layout, and (**c**) multilayer structure of the MSRR surface MIMO antenna array.

**Fig. 3:**
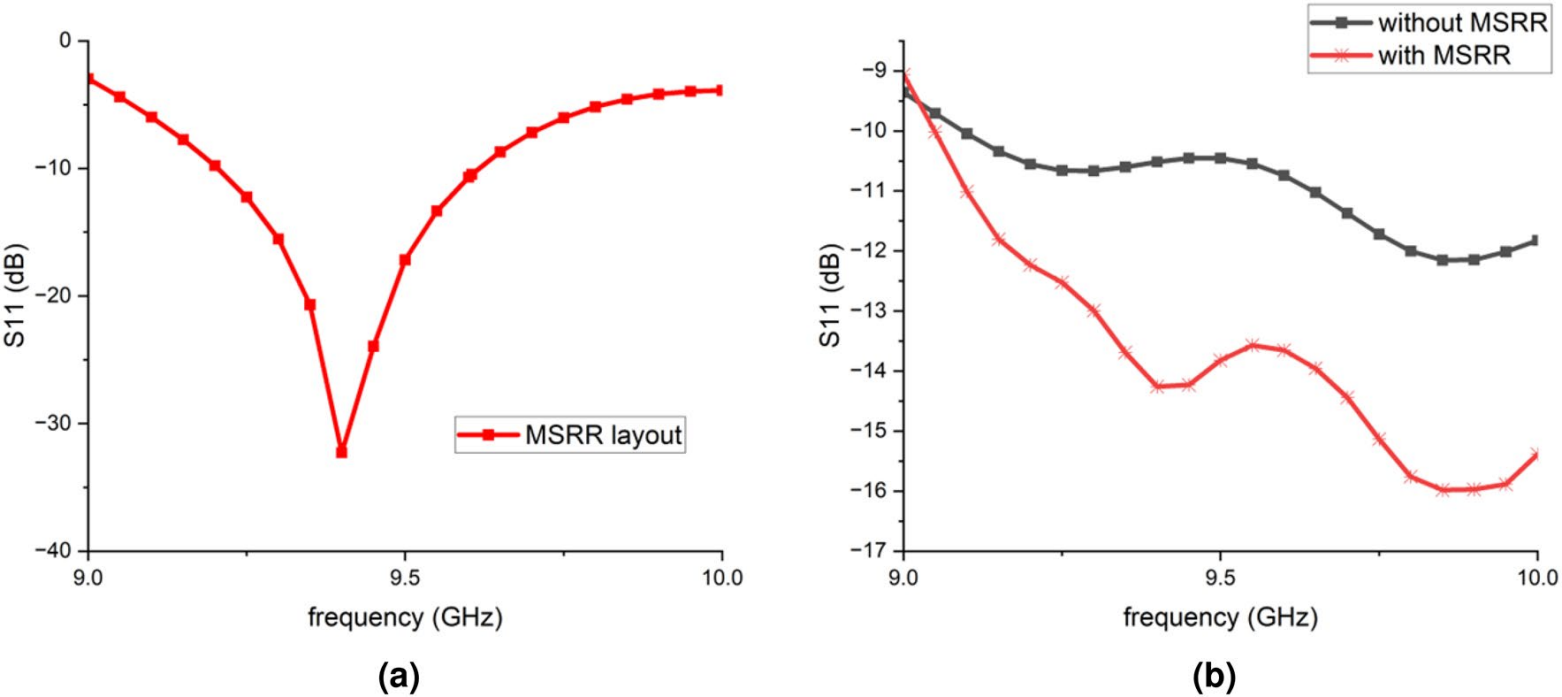
Reflection coefficients: (**a**) $S_{11}$ of the MSRR surface layout; (**b**) $S_{11}$ of the antenna array with and without the MSRR metasurface.

**Fig. 4:**
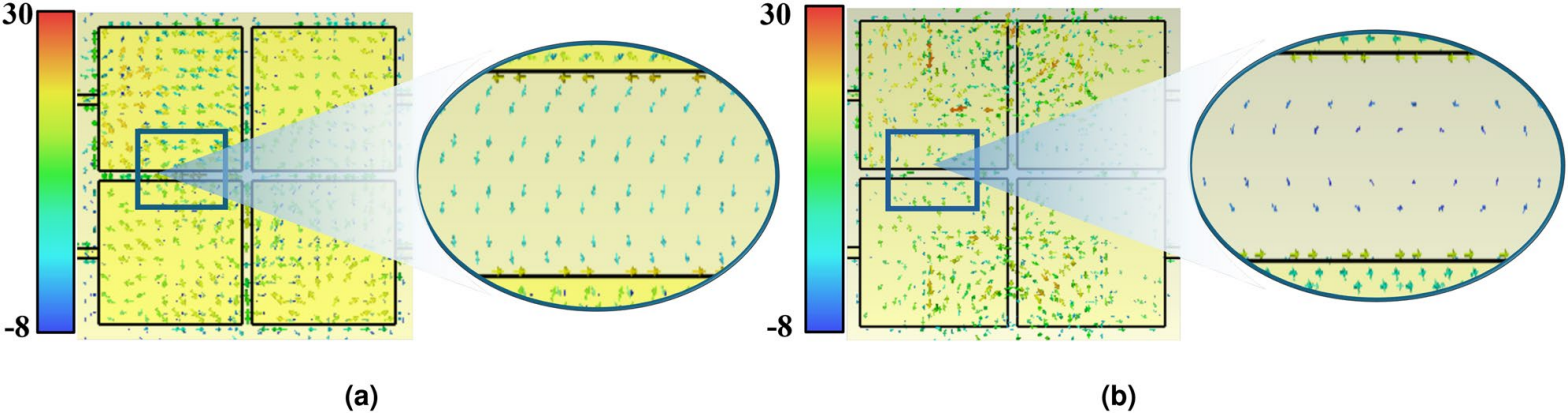
Simulated surface-current distribution on the $2\times 2$ array at $f=9.90$ GHz (representative notch frequency), when Antenna 1 is excited and the remaining ports are terminated with 50 $\Omega$: (**a**) without the MSRR metasurface; (**b**) with the MSRR metasurface. The arrow vectors indicate current direction and the color scale indicates current magnitude. The element/port indexing follows Fig. 2a (Antenna 1: top-left, Antenna 2: bottom-left, Antenna 3: top-right, Antenna 4: bottom-right), and the color bar shows the surface-current magnitude in dB (A/m).

Figure 1 shows two types of MSRRs which are used to form the metasurface in this study. The inner rings comprise traditional split-ring resonators, while the outer rings are composed of two square rings. The cross introduces additional current paths and parasitic capacitances, enabling fine-tuning of the resonance frequency and bandwidth. Compared with prior SRR-based decoupling metasurfaces that typically employ a single-loop (or single-type) SRR unit cell to form a narrow rejection band, the proposed MSRR introduces extra tuning degrees of freedom by combining multi-ring loading (two outer square rings) and a cross-loading feature. These modifications provide additional current paths and effective capacitive/inductive loading, which facilitates resonance alignment and multi-mode behavior within a compact footprint. Furthermore, two MSRR variants (Fig. 1) are combined in the 2 $\times$ 3 metasurface layout, forming a composite rejection response over the 9–10 GHz band. This design choice is consistent with the measured broadband coupling suppression in Fig. 6 and the deeper notches observed near the band edges, which are characteristic of resonant metasurface decoupling. This contributes to the ability of metasurface to operate efficiently across the desired 9–$10~\textrm{GHz}$ band and supports the formation of multiple resonant modes when combined with the outer square rings. The resonant frequencies of the MSRR are influenced by various characteristics, such as cleavage width, gap size and its position, and metal strip width. The geometric details of the MSRRs are listed in Table 1. Figure 3a illustrates simulated $S_{11}$ for the metasurface consisting of these two MSRRs as shown in Fig. 2b. The distance between each element is $12.6~\textrm{mm}$.

As a proof of concept, a 2 $\times$ 2 MIMO antenna array incorporated with 2 $\times$ 3 MSRR metasurface operating in the frequency range from 9 to 10 GHz, as illustrated in Fig. 2a,b, has been designed, fabricated, and characterized. The antenna array comprises a ground plane, a FR4 substrate with a dielectric constant of 4.3, and a copper patch antenna. The patch antenna is energised by microstrip lines. The FR4 substrate thickness is of 1.5 mm, the microstrip line width is 1 mm, and the antenna patch elements are uniformly and linearly arranged on the substrate, with a separation of 1 mm between the elements. The MSRR metasurface is positioned directly above the MIMO antenna array, as illustrated in Fig. 2c. Rogers4003C is selected as the substrate for metasurface. Each MSRR element on this substrate is precisely manufactured so to effectively resonate within the target frequency band, thereby ensuring an effective radiation pattern and resonant behavior.

Figure 3b shows simulated |$S_{11}$| of the antenna array with and without MSRR surface. It can be observed that after integrating the MSRR structure, the impedance matching of the antenna is improved. Figure 4 illustrates the simulated surface current distributions of the antenna array with and without the MSRR structure. In the proposed JCAS system configuration, Antennas 1 and 3 are designated as the transmitting (communication) elements, whereas Antennas 2 and 4 serve as the receiving (sensing) elements (see Fig. 2a). Therefore, the mutual coupling between the transmitting and receiving arrays is primarily characterized by the S-parameters $S_{12}$, $S_{14}$, $S_{32}$, and $S_{34}$. Surface current distributions on the array are illustrated in Fig. 4. When Antenna 1 is excited, without the MSRR surface, Antennas 2 and 4 experience dense and notably high surface current, most notably along the edges that face Antenna 1, revealing a pronounced leakage path formed by surface-wave and slot-field coupling. Return currents are also visible along the inter-element gaps, indicating that common-substrate surface modes participate in the coupling. With the MSRR integrated, on the other hand, both the magnitude and the spatial extent of the surface current on Antennas 2 and 4 are markedly reduced. The previously continuous high-magnitude bands along the facing edges break into sparse, low-magnitude; the interior current pattern changes from large connected regions to localized residuals. The current corridor across the element gaps is interrupted, which indicates strong suppression of surface-wave propagation and of the near-field coupling path. Energy remains confined around the excited Antenna 1 and no longer couples efficiently to the receive subarray. These observations confirm that the MSRR reshapes the surface-current and near-field distributions on the array aperture, weakens the substrate-supported surface and slot modes, and mitigates the principal coupling route between the communication and sensing elements.

## Simulation and measurement results

**Fig. 5:**
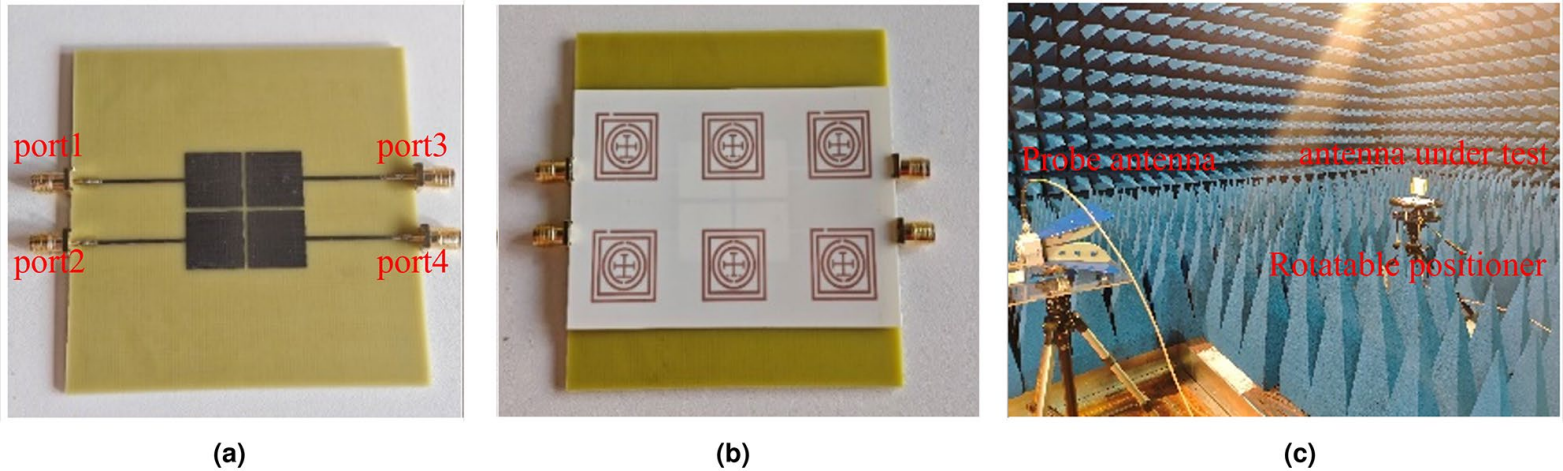
Photographs of the fabricated prototypes and measurement setup: (**a**) fabricated $2\times 2$ patch array on FR4 with four SMA ports (port indexing follows Fig. 2a: Ports 1 and 3 are the TX ports, and Ports 2 and 4 are the RX ports); (**b**) the assembled prototype with the $2\times 3$ MSRR metasurface superstrate (printed on Rogers 4003C) aligned above the patch array as illustrated in Fig. 2c; (**c**) anechoic-chamber measurement configuration, showing the antenna-under-test mounted on a rotatable positioner and the linearly polarized probe antenna. The multi-port S-parameters were measured using a calibrated VNA via coaxial connections, and the radiation patterns were measured by rotating the antenna-under-test.

Photos of the fabricated antenna array and the integrated MSRR structure are presented in Fig. 5. Experimental measurements were conducted as follows: the multi-port S-parameters were measured using a vector network analyzer (VNA), and the radiation patterns were measured in an anechoic chamber. To improve reproducibility, the measurement procedure is detailed as follows. The S-parameters were measured using a VNA after standard calibration at the coaxial cable ends. When measuring a specific coupling coefficient between two ports, the corresponding ports were connected to the VNA, while all remaining antenna ports were terminated with matched 50 $\Omega$ loads. This procedure was repeated for the relevant port pairs to obtain the mutual-coupling coefficients (e.g., $S_{12}$, $S_{14}$, $S_{32}$, and $S_{34}$) and the reflection coefficients of the antenna elements for both configurations (with and without the MSRR metasurface). Note that the beamforming weights are not physically applied during the VNA measurements. Instead, the measured S-parameters are used to form the frequency-selective SI coupling channel matrix $H_{\textrm{SI}}(f)$ (TX ports: 1 and 3; RX ports: 2 and 4). The multi-frequency NSP beamforming weights are then computed and applied in post-processing, and the effective coupling metric and SINR are evaluated using Eqs. (5)–(7). Therefore, the “JCAS coupling performance” and “SINR versus radar/sensing-beam angle” results in Figs. 7, 8 and 9 are obtained by applying the computed weights to the measured coupling matrix in post-processing, rather than by a hardware beamforming network during measurement.

Radiation patterns were measured in the anechoic chamber with the antenna-under-test mounted on a rotatable positioner and a linearly polarized probe antenna. In the pattern measurement reported in Fig. 11, Antenna 1 was excited while the other ports were terminated with 50 $\Omega$ loads, and the co-polarized E-plane pattern at 9.5 GHz ($\theta =90^\circ$) was recorded. In addition, the angle-dependent evaluation range corresponds to typical forward-sector sensing scenarios (e.g., forward-looking vehicular radar and indoor directional sensing) and aligns with the practical constraints of the array and measurement setup. This element-pattern measurement is used to verify that the MSRR metasurface does not degrade the radiation characteristics; beam-weighted array patterns are evaluated based on the coupling matrix and beamforming formulation rather than being directly measured by an analog beamforming network.

**Fig. 6:**
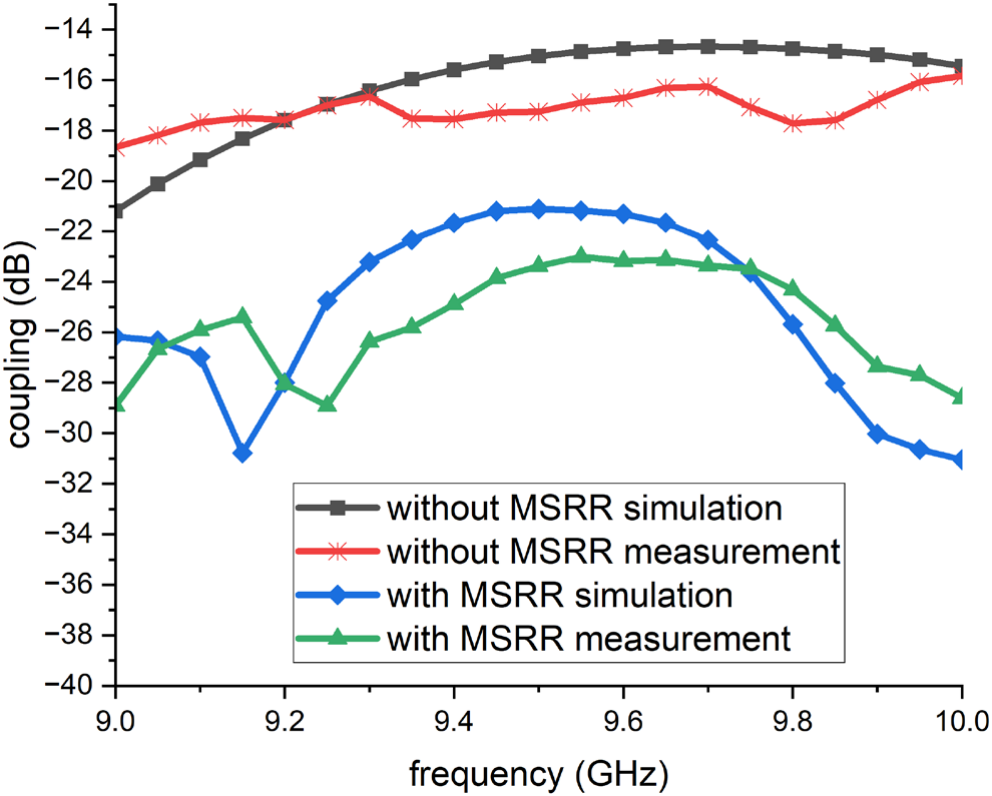
Simulated and measured coupling between the co-located TX/RX arrays versus frequency in the non-JCAS configuration, with and without the MSRR metasurface.

**Fig. 7:**
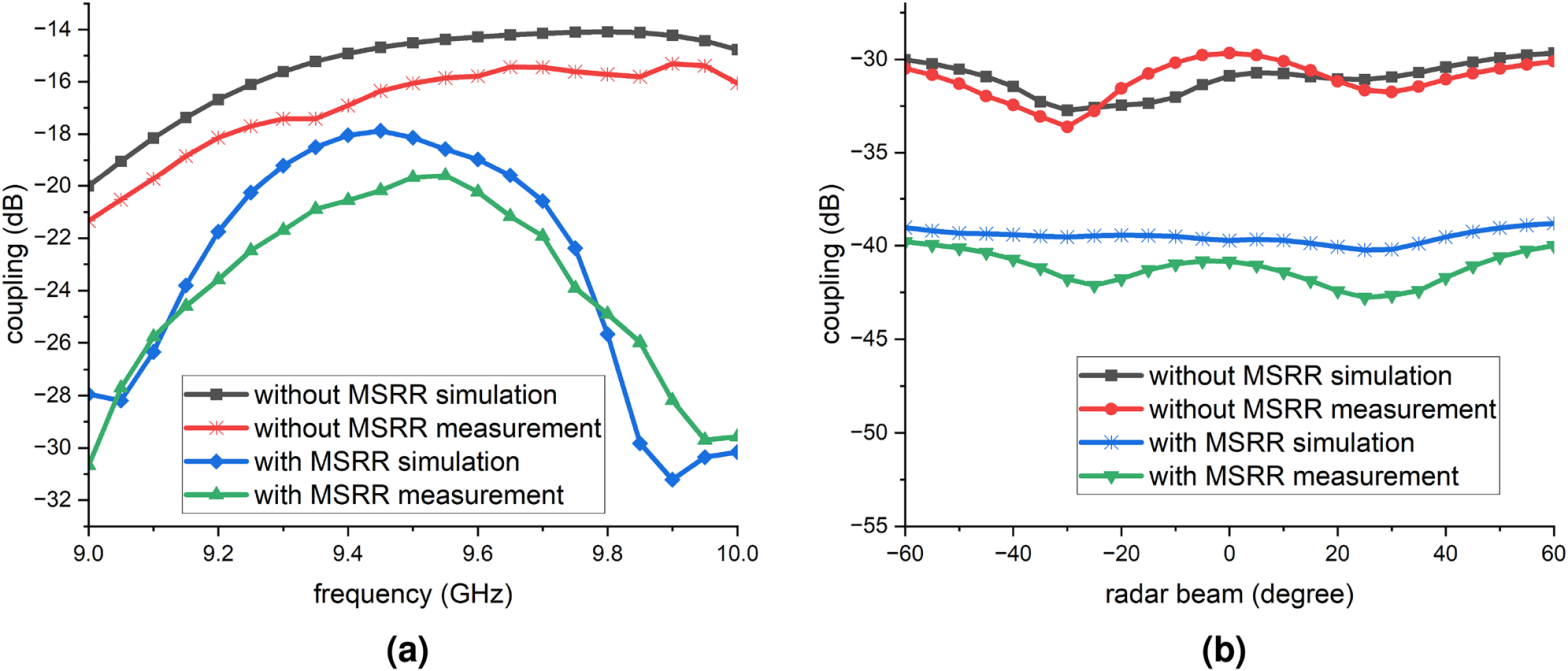
JCAS system with 0 frequency null coupling performance (computed using Eq. (6) with NSP-derived beamforming weights applied in post-processing based on the measured coupling matrix): (**a**) versus frequency; (**b**) versus radar-beam scan angle.

**Fig. 8:**
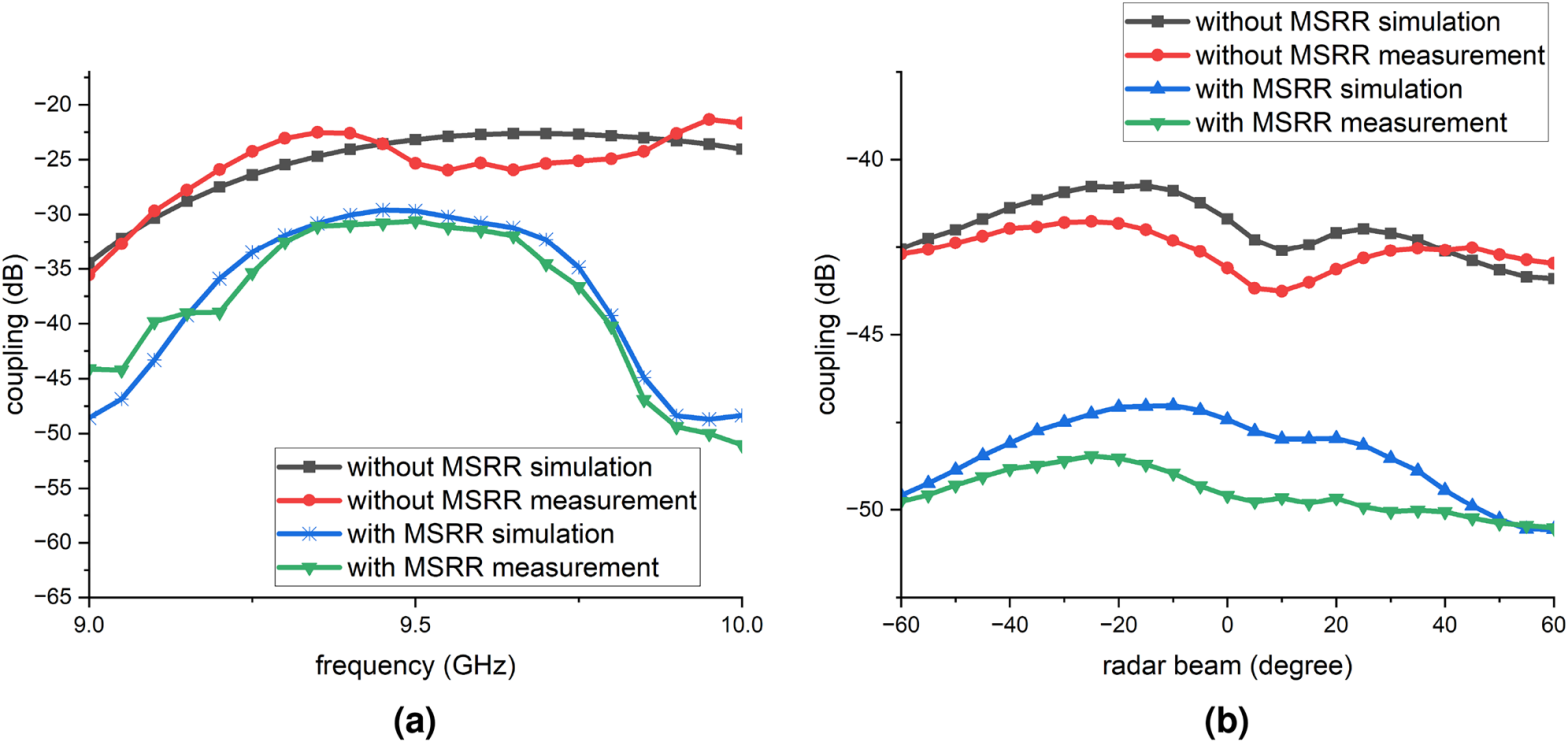
JCAS system with 1 frequency null coupling performance (computed using Eq. (6) with NSP-derived beamforming weights applied in post-processing based on the measured coupling matrix): (**a**) versus frequency; (**b**) versus radar-beam scan angle.

**Fig. 9:**
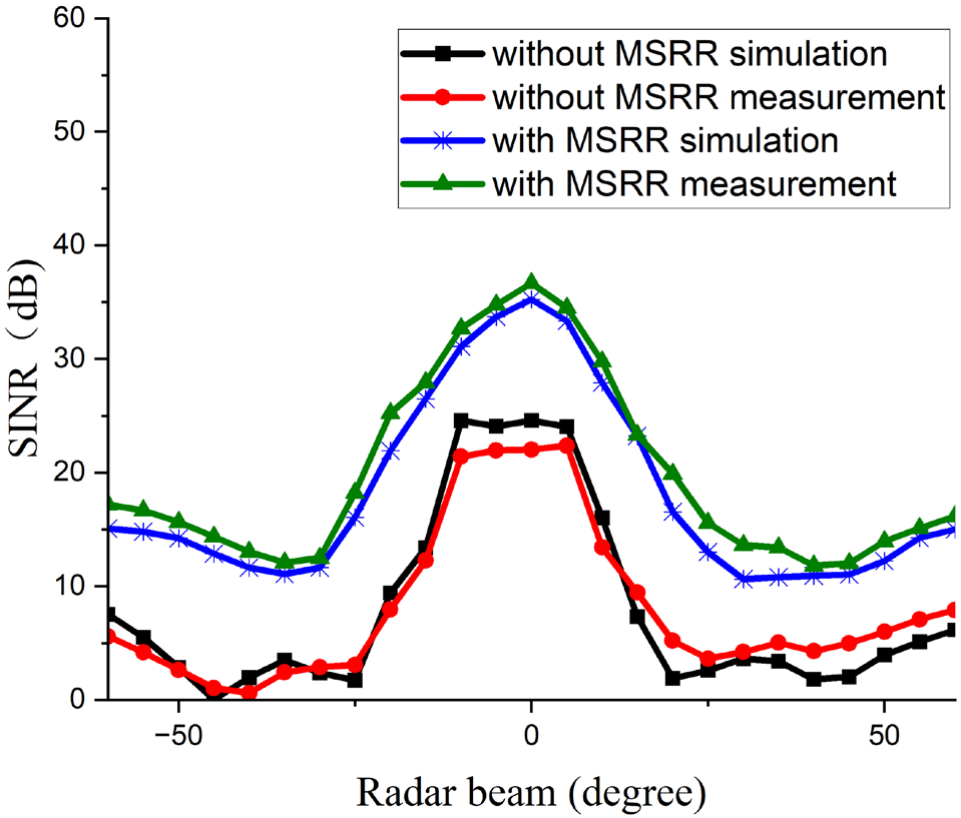
SINR versus radar-beam scan angle for the JCAS configuration with 0 frequency null, computed using Eq. (7) from the measured coupling channel matrix and the corresponding beamforming weights.

**Fig. 10:**
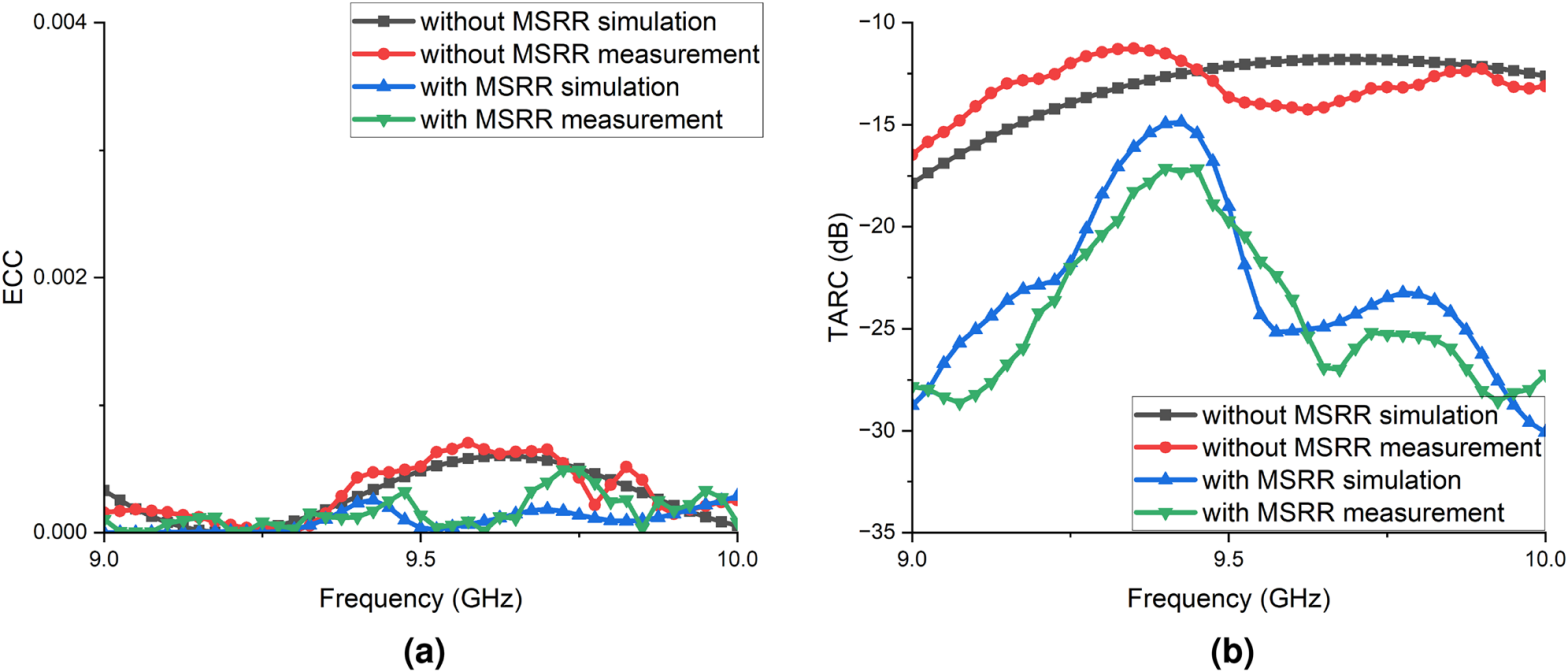
Simulated and measured performance of the $2\times 2$ JCAS array with and without the MSRR metasurface: (**a**) envelope correlation coefficient (ECC); (**b**) total active reflection coefficient (TARC).

**Fig. 11:**
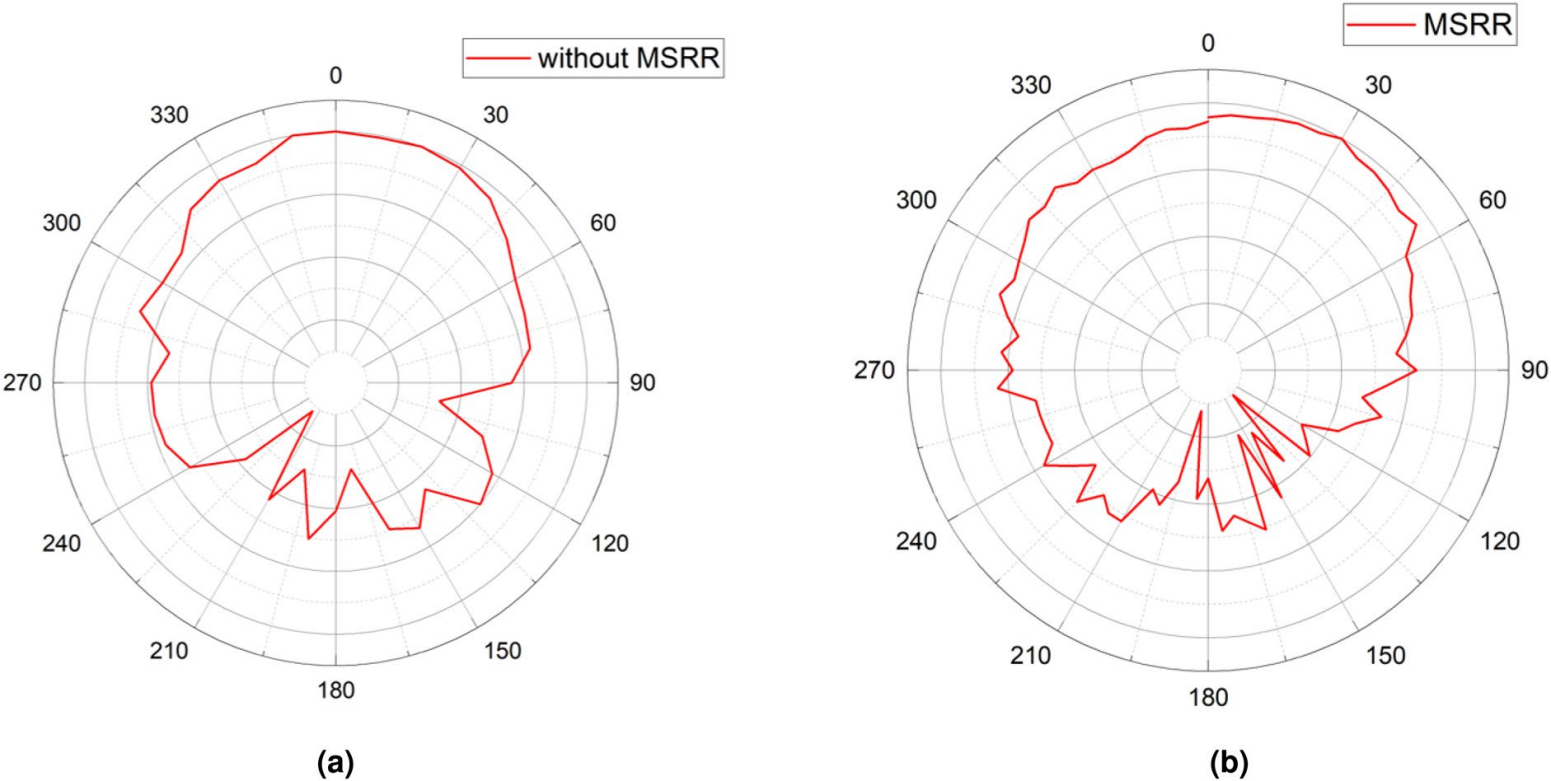
Measured co-polarized E-plane ($\theta =90^\circ$) radiation patterns at 9.5 GHz for single-port excitation of Antenna 1 (other ports terminated with 50 $\Omega$): (**a**) without the MSRR structure; (**b**) with the MSRR structure.

Figure 6 presents the coupling results obtained from a non-JCAS system for simulated and measured responses both with and without MSRR metasurface. Without the MSRR metasurface, the simulated (black squares) remaining around $-22$ to $-18~\textrm{dB}$ across the band. When the MSRR metasurface is implemented, the simulated (blue diamonds) and measured (green triangles) curves exhibit a clear reduction in coupling. Specifically, isolation improves to $-28$ to $-34~\textrm{dB}$ in the 9.0–$9.2~\textrm{GHz}$ region, maintains a relatively flat $-22$ to $-26~\textrm{dB}$ plateau in the 9.2–$9.6~\textrm{GHz}$, and remains better than $-26~\textrm{dB}$ up to $10~\textrm{GHz}$. The deepest notches of simulation results occur near $9.15~\textrm{GHz}$ and $10~\textrm{GHz}$, where the measured isolation reaches about $-34~\textrm{dB}$, indicating that the MSRR surface effectively suppresses coupling hot-spots at the band edges. The agreement between simulation and measurement is within 1–$3~\textrm{dB}$, with slight frequency shifts at the isolation minima. The very deep isolation minima observed at specific frequencies (e.g., around 9.9 GHz) are attributed to the resonant nature of the MSRR metasurface and its interaction with the dominant near-field coupling path between the co-located TX/RX arrays. For closely spaced patch arrays on a common substrate, the TX–RX leakage is mainly carried by a combination of fringing near fields and a surface-current corridor on the metallization/ground region between the elements.

The MSRR acts as a frequency-selective resonant scatterer. Physically, the current loop along the ring traces provides an effective inductance, while the splits/gaps introduce capacitance, forming an LC-type resonance. Near a resonant mode, the induced currents on the MSRR become strong and the metasurface produces a pronounced scattered near field with rapidly varying phase. As a result, the net coupled field at the RX ports can be interpreted as the superposition of (i) the direct near-field leakage from the TX array and (ii) the MSRR-scattered contribution. At the notch frequency, the scattered contribution becomes comparable in magnitude and approximately out of phase along the dominant coupling corridor, leading to destructive interference and a sharp reduction of the effective coupling. This mechanism is consistent with the simulated surface-current distributions in Fig. 4 (taken at a representative notch frequency). Without the MSRR, a continuous current corridor is observed between the excited TX element and the adjacent elements, which facilitates strong TX–RX leakage. With the MSRR, the current corridor is significantly weakened/broken and the induced currents on the receiving elements are suppressed, indicating that the resonant metasurface effectively blocks and/or cancels the dominant coupling path. Therefore, very deep notches occur only at the frequencies where the MSRR resonance most effectively reshapes the near field and satisfies the cancellation condition.

The effectiveness of the proposed MSRR surface decoupling structure was further evaluated in the context of the JCAS system. The corresponding simulation and measurement results are presented in Fig. 7. Figure 7a shows the coupling across 9–$10~\textrm{GHz}$ in a JCAS scenario that includes a zero frequency null. Without the MSRR metasurface, the simulation baseline increases smoothly from about $-20~\textrm{dB}$ at $9.00~\textrm{GHz}$ to around $-15~\textrm{dB}$ over 9.6–$10.0~\textrm{GHz}$. With the MSRR, the curves shift downward. Around $9.05~\textrm{GHz}$ the simulated coupling is $-28.5~\textrm{dB}$ and across 9.3–$9.6~\textrm{GHz}$ it remains $-19$ to $-20~\textrm{dB}$; and at $9.90~\textrm{GHz}$ a sharp minimum reaches $-31.5~\textrm{dB}$. At $10.00~\textrm{GHz}$ the level remains $\le -30~\textrm{dB}$. Comparing to the no-MSRR metasurface baseline, the smallest improvement is about $3~\textrm{dB}$ at $9.4~\textrm{GHz}$ and the largest is about $14~\textrm{dB}$ at $9.9~\textrm{GHz}$. Simulation and measurement exhibit highly consistent shapes and extremum locations, with differences mainly in magnitude, typically 1–$3~\textrm{dB}$.

Given that the JCAS system differentiates between communication and radar/sensing beams, and the radar/sensing beam is able to detect the environment by changing the angle of the beam, thus the coupling performance were also evaluated within a radar/sensing angular range from $-60^{\circ }$ to $60^{\circ }$, due to its targeted application scenario. Specifically, many practical JCAS use cases, such as forward-looking vehicular radar or indoor directional sensing, require only unidirectional beam coverage in the forward sector^33^. This study adopts a unidirectional evaluation range from $-60^{\circ }$ to $60^{\circ }$, which is sufficient for assessing the decoupling performance of the MSRR metasurface structure in the intended beamforming direction. This range also aligns with the practical constraints of the antenna array and measurement platform. Considering the average coupling between 9 to $10~\textrm{GHz}$ at each single radar angle, and the results are shown in Fig. 7b. Without the MSRR metasurface, the simulated and measured traces lie between $-30$ and $-34~\textrm{dB}$, with a minimum near $-25^{\circ }$ radar/sensing beam, where the measurement reaches approximately $-34~\textrm{dB}$ and the simulation about $-33~\textrm{dB}$. At the scan limits $\pm 60^{\circ }$ the coupling reaches to roughly $-30~\textrm{dB}$. With the MSRR surface, the coupling level shifts downward across the full angular span. The simulated response is nearly flat around $-40~\textrm{dB}$, while the measured response shows its deepest notch near $+25^{\circ }$ at about $-43~\textrm{dB}$ and rises toward $-40~\textrm{dB}$ at the edges. Comparing to the case without MSRR metasurface, the isolation improvement is, in average, about 9 to $11~\textrm{dB}$ over the scan, approaching $11~\textrm{dB}$ near the measured notch at $+25^{\circ }$, and about 8 to $10~\textrm{dB}$ at $\pm 60^{\circ }$. The simulated and measured results agree closely in waveform shape and in the locations of extrema, and the remaining amplitude differences are typically within $2~\textrm{dB}$.

Figure 8a presents the frequency dependency of the coupling in a JCAS configuration with a single frequency null. Without the MSRR metasurface, the simulation is about $-35~\textrm{dB}$ at $9.00~\textrm{GHz}$ and remains near $-25~\textrm{dB}$ from 9.5 to $9.9~\textrm{GHz}$; the measurement is about $-35~\textrm{dB}$ at $9.00~\textrm{GHz}$ and rises to $-25~\textrm{dB}$ at $9.4~\textrm{GHz}$. With the MSRR surface, the coupling decreases markedly: at $9.00~\textrm{GHz}$ the simulation is about $-49~\textrm{dB}$ and the measurement about $-45~\textrm{dB}$; from 9.3 to $9.6~\textrm{GHz}$ both form a plateau around $-35$ to $-37~\textrm{dB}$; from 9.90 to $9.95~\textrm{GHz}$ the deepest notch appears, with a simulated minimum near $-50~\textrm{dB}$ and a measured minimum near $-52~\textrm{dB}$; at $10.00~\textrm{GHz}$ the level remains close to $-50~\textrm{dB}$. Comparing to the baseline without MSRR metasurface, the isolation improvement at $9.00~\textrm{GHz}$ is about $14~\textrm{dB}$ in simulation and about $10~\textrm{dB}$ in measurement; at $9.50~\textrm{GHz}$ the improvement is about $7~\textrm{dB}$ in simulation and about $8~\textrm{dB}$ in measurement; at $9.90~\textrm{GHz}$ the improvement is about $25~\textrm{dB}$ in simulation and about $27~\textrm{dB}$ in measurement. Simulation and measurement agree well in curve shape and in the locations of extrema. Overall, the MSRR lifts the isolation from a range of $-25~\textrm{dB}$ to $-35~\textrm{dB}$ to the range of $-35$ to $-50~\textrm{dB}$, with the most pronounced improvement from 9.9 to $10.0~\textrm{GHz}$.

Figure 8b shows the coupling versus radar/sensing-beam angle from $-60^{\circ }$ to $+60^{\circ }$ for a JCAS configuration with a single frequency null. Without the MSRR metasurface, the simulated and measured traces lie between $-41$ and $-44~\textrm{dB}$, with a broad minimum near $10^{\circ }$ where the measurement approaches about $-42~\textrm{dB}$ and the simulation about $-44~\textrm{dB}$; at $\pm 60^{\circ }$ they reach roughly $-42~\textrm{dB}$. With the MSRR metasurface, the coupling is getting lower, remaining between about $-47$ and $-50~\textrm{dB}$ across the whole scan. The simulated response is closest to $-47~\textrm{dB}$ near $-20^{\circ }$, approaching $-50~\textrm{dB}$ at the edges, while the measured response follows the same trend and reaches nearly $-50~\textrm{dB}$ near $+60^{\circ }$. The MSRR metasurface therefore provides an essentially isolation gain of approximately 5 to $8~\textrm{dB}$ over the entire scan, and up to $8~\textrm{dB}$ at $\pm 60^{\circ }$. Simulation and measurement agree closely in both curve shape and extremum angles; the remaining magnitude differences are typically within 1 to $2~\textrm{dB}$.

To place the proposed MSRR-assisted decoupling in context, Table 2 benchmarks representative mutual-coupling mitigation techniques reported in the literature. DGS- and EBG-based approaches can suppress surface-wave coupling by modifying the ground plane or introducing periodic band-gap cells, typically achieving a moderate reduction in mutual coupling. More aggressive three-dimensional decoupling walls (e.g., mushroom-based structures) can provide very high isolation, but at the cost of increased profile and mechanical complexity. Metasurface-superstrate solutions have also been reported to improve isolation and/or bandwidth, and some are designed with scalability to larger arrays in mind. Compared with these antenna-only decoupling studies, our MSRR metasurface is a planar, fabrication-friendly add-on for the patch array, and we further demonstrate a hardware–algorithm co-optimization for a JCAS full-duplex architecture: the metasurface reduces the coupling channel, while the multi-frequency NSP beamforming suppresses the effective SI in the JCAS configuration. Since the listed works involve different antenna types, bands, and array sizes, the values in Table 2 should be interpreted as a benchmark rather than a strict one-to-one ranking. A key limitation of the proposed MSRR is its resonant nature, implying that the unit geometry needs to be re-tuned when the operating band or the antenna platform changes.

**Table 2:** Comparison of representative decoupling techniques reported in the literature and this work.

Ref.	Decoupling approach	Antenna configuration	Band	Reported isolation/coupling reduction	Notes/trade-offs
^13^	Defected ground structure (DGS)	Collocated 4-port MIMO (patch + ring patch)	2.3–2.6 GHz	Isolation improvement of 7 dB; mutual coupling as low as $-25$ dB	Requires ground modification; mainly mitigates surface-wave coupling
^12^	Dual-bandgap stacked EBG (VBS-EBG)	Air-gap based dual-band MIMO patch	Dual-band (5G)	Mutual coupling reduction of 16.16 dB/6.05 dB at the first/second resonance	Periodic EBG concept; performance tied to stop-band design
^16^	CSR-loaded mushroom-type EBG	Two E-plane coupled microstrip antennas	2.2–2.7 GHz	Mutual coupling reduction of 8.13 dB; 48.9% miniaturization vs. conventional mushroom EBG	Compact EBG unit; still relies on periodic resonance behavior
^14^	Double-layer metamaterial mushroom wall	4-element SICBS MIMO system	2.396–2.45 GHz	Isolation enhancement of 16 dB (parallel-directed pairs)	Strong isolation but 3D wall increases profile/assembly complexity
^32^	SRR metasurface cover (MAAD)	Two-element closely spaced patch MIMO	5.8 GHz	Isolation increased from $\sim$8 dB to >27 dB; $|S_{11}|<-15$ dB bandwidth 360 MHz $\rightarrow$ 900 MHz	Superstrate-type metasurface; improves both isolation and matching bandwidth
^31^	$\epsilon$-negative metasurface superstrate	4 $\times$ 4 antenna array	5.4–6.2 GHz	The isolation between elements is increased from around 8 dB to more than 25 dB within the band of interest	Designed with scalability to larger arrays; requires additional superstrate layer
This work	Planar MSRR metasurface + multi-frequency NSP (hardware–algorithm co-optimization)	2 $$\times$$ 2 TX/RX patch arrays for JCAS	9–10 GHz	Metasurface-only: measured coupling improves from $$\sim (-22$$ to $$-18)$$ dB to $$\le -26$$ dB (min. $$\sim -34$$ dB). JCAS (1 null): measured coupling improves to $$\sim (-35$$ to $$-50)$$ dB (min. $$\sim -52$$ dB)	Planar add-on and fabrication-friendly; algorithm further suppresses effective SI. Resonant unit needs re-tuning for other bands/geometries

**Table 3:** Benchmarking representative SRR/metasurface-based decoupling works and this work (values are reported in the corresponding references).

Ref.	SRR/metasurface type	Antenna configuration	Band	Reported isolation/coupling performance	Key notes (distinction to this work)
^32^	Suspended metasurface superstrate with periodic square SRRs (negative-$\mu$ band)	Two-element patch array, extremely small spacing	$\sim$5.8 GHz	Isolation increased from $\sim$8 dB to >27 dB; $|S_{11}|<-15$ dB bandwidth 360 MHz $\rightarrow$ 900 MHz	Classic SRR-superstrate decoupling for 1$\times$2 MIMO; requires suspended superstrate and matching re-tuning
^31^	Double-layer metasurface superstrate (negative-$\epsilon$ over wide angles)	4$\times$4 patch array	5.4–6.2 GHz	The isolation between elements is increased from around 8 dB to more than 25 dB within the band of interest	Focus on scalability to larger arrays; multi-layer superstrate
^34^	Coupled metamaterial slabs using interdigital particles + SRRs	Dual-polarized patch antennas (radiation-leakage coupling)	1.95–2.2 GHz	$\sim$ 7 dB isolation improvement over 1.95–2.2 GHz; compact volumetric module	Hybrid MTM device; targets radiation-leakage coupling path
^35^	Coplanar metasurface using multilayer hybrid SRR meta-atoms (single-negative)	Wideband coupled patch MIMO arrays (2-element/linear/2D)	Example around 5 GHz	Mutual coupling suppressed to $<-20$ dB; bandwidth improved (e.g., 21.7%$\rightarrow$25.4%)	Wideband decoupling and no profile increase; focuses on conventional MIMO arrays
This work	Single-layer MSRR superstrate (two MSRR variants; outer rings + cross-loading) + multi-frequency NSP	2$$\times$$2 co-located TX/RX patch arrays for JCAS	9–10 GHz	Passive (non-JCAS): coupling improved from $$\sim (-22$$ to $$-18)$$ dB to $$\le -26$$ dB (min. $$\sim -34$$ dB). JCAS (1 null): measured coupling down to $$\sim -52$$ dB (max improvement $$\sim$$27 dB)	Distinctive contribution: modified SRR geometry (multi-mode tuning) and hardware–algorithm co-optimization for JCAS SI suppression (metasurface + NSP)

Table 3 shows that SRR/metasurface-based decoupling has been widely explored for conventional MIMO arrays, often with two-element configurations or with different structural trade-offs (e.g., suspended superstrates, multi-layer superstrates, or coplanar inserts). In contrast, our MSRR design targets the co-located TX/RX SI channel in a JCAS full-duplex architecture. The modified SRR geometry (outer rings and cross-loading) provides additional tuning degrees of freedom to form a composite rejection behavior over the 9–10 GHz band with a compact 2 $\times$ 3 metasurface, and the effective SI is further reduced by the multi-frequency NSP beamforming framework. Therefore, the advantage of this work is not only the coupling suppression by a modified SRR metasurface, but also the system-level hardware–algorithm co-optimization validated by measurement. Although only a 2 $\times$ 2 array is experimentally validated, it should be noted that a 2 $\times$ 2 finite array contains no interior elements and all elements are edge elements. Therefore, the simulated and measured multi-port coupling results reported in this work are obtained under a fully non-periodic, edge-dominated configuration, which inherently includes edge scattering and edge-induced coupling.

To make the hardware–algorithm co-design effect explicit, he contributions of the MSRR metasurface and NSP beamforming are summarized in Table 4 using the measured coupling results in Figs. 6, 7 and 8. The MSRR metasurface provides a clear reduction of the effective coupling in both the 0-null and 1-null JCAS configurations, while the NSP frequency-null constraint further suppresses the residual SI compared with the 0-null baseline. The combined MSRR+NSP configuration achieves the lowest effective coupling over the entire scan range.

**Table 4:** Contributions of the MSRR metasurface (hardware) and NSP beamforming (algorithm) based on measured results in Figs. 6, 7 and 8.

Configuration	MSRR	Nulls	Effective coupling (dB)
Hardware only (Fig. 6)	Yes	0	$\approx -22$ to $-32$
JCAS baseline beamforming (Fig. 7b red and black lines)	No	0	$\approx -30$ to $-34$
Hardware +JCAS baseline beamforming (Fig. 7b blue and green lines)	Yes	0	$\approx -40$ to $-42$
Hardware + Algorithm (Fig. 8b blue and green lines)	Yes	1	$\approx -47$ to $-50$

The SINR performance of the JCAS system, as a function of radar/sensing-beam angle from $-60^{\circ }$ to $+60^{\circ }$, without frequency nulls has also been analyzed, with the corresponding results illustrated in Fig. 9. As the MSRR reduces the SI coupling between TX and RX arrays, the interference term in the SINR denominator decreases accordingly, resulting in the observed SINR improvement across the scan angles. Without the MSRR metasurface, the SINR remains below $25~\textrm{dB}$ across the scan angles. The simulation gives roughly 4 to $6~\textrm{dB}$ near $\pm 60^{\circ }$, falls to about 0 to $2~\textrm{dB}$ around $-45^{\circ }$, and rises to a broad plateau of approximately $23~\textrm{dB}$ between $-10^{\circ }$ and $+10^{\circ }$. The measurement follows the same profile, reaching about 20 to $21~\textrm{dB}$ between $-10^{\circ }$ and $+10^{\circ }$, and has around 1 to $3~\textrm{dB}$ difference with the simulation results near $\pm 60^{\circ }$. With the MSRR metasurface, the SINR increases markedly over the entire scan angles. The measurement exhibits the same bell-shaped trend as the simulated, reaching peak of about $37~\textrm{dB}$ at $0^{\circ }$, maintaining $30~\textrm{dB}$ from about $-15^{\circ }$ to $+15^{\circ }$, and yielding $12~\textrm{dB}$ near $\pm 30^{\circ }$. The improvement is therefore substantial: at $0^{\circ }$ the improvement is about $14~\textrm{dB}$ and at $\pm 60^{\circ }$ the improvement is around $10~\textrm{dB}$. Simulation and measurement agree closely in waveform shape and in the locations of the peak and the side minima. The measured values are generally within 1 to $3~\textrm{dB}$ of the simulated, Minor angular shifts of a few degrees are visible.

Figure 10a presents the envelope correlation coefficient (ECC) of the $2\times 2$ antenna array over 9.0–10.0 GHz, comparing simulation and measurement results with and without the MSRR metasurface. Without the metasurface, a modest rise appears at 9.4–9.7 GHz, with a simulated peak of approximately 0.001 and a measured peak of 0.001. With the metasurface, the ECC remains essentially near zero, with both simulation and measurement $\le 0.001$, showing only shallow undulations around 9.55–9.7 GHz. It is worth to emphasize that the ECC across the entire band is far below the commonly used 0.05 guideline, indicating very low inter-port correlation and excellent diversity performance. Relative to the case without the metasurface, a reduction of approximately 3–5 times is maintained in this frequency region, confirming the decorrelation and isolation enhancement provided by the MSRR surface.

Figure 10b shows the total active reflection coefficient (TARC) of the $2\times 2$ JCAS array over 9.0–10.0 GHz for configurations with and without the MSRR metasurface, in both simulation and measurement. Without the metasurface, the TARC remains relatively high across the band, approximately from $-16$ to $-12$ dB. With the metasurface, the TARC is markedly reduced to about $-30$ to $-18$ dB, with pronounced minima near 9.60 GHz (approximately $-26$ dB in the simulation and $-27$ dB in the measurement) and another low near 9.95–10.00 GHz (approaching $-30$ dB in the simulation and about $-28$ dB in the measurement). Relative to the case without the metasurface, this corresponds to an improvement in active matching of approximately 10–15 dB over most of the band, confirming that the MSRR metasurface effectively lowers the TARC and enhances active power transfer in the JCAS array.

The differences of almost all experimental and simulation data are within the range of 1 to 3 dB. These discrepancies between the simulated and the measured can be attributed to fabrication tolerances in the MSRR metasurface geometry and substrate permittivity. Also the small air gaps caused by imperfect lamination between the MSRR metasurface layer and the antenna layer can bring the difference of simulation and measurement results.

The radiation pattern of Antenna 1 was experimentally characterized, and the results are presented in Fig. 11, illustrating the radiation patterns of the antenna array with and without MSRR metasurface decoupling structure. Without the MSRR surface, the forward hemisphere (approximately $-60^{\circ }$ to $+60^{\circ }$) exhibits a main lobe with noticeable edge ripple, and the backward hemisphere shows multiple back-radiation features and shallow sidelobes, producing a serrated pattern that indicates persistent surface-wave channels and coupling hot spots between adjacent elements. With the MSRR metasurface, sidelobe formation is pushed outward in angle, which expands the effective forward main-beam angular sector. Correspondingly, reducing the likelihood of coupling toward those directions. In addition, the suppression of sidelobes in specific directions lowers susceptibility to lateral interference and multipath reflections. These observations indicate that the MSRR structure not only mitigates mutual coupling but also improves the overall radiation performance of the antenna array in the JCAS system.

## Conclusion

This work focuses on the antenna mutual coupling suppression in the JCAS and proposes a metamaterial decoupling structure based on the MSRR. It introduces hardware-algorithm collaborative scheme to achieve efficient decoupling and communication link enhancement within the 9–10 GHz frequency band. Through CST full-wave simulation and experimental verification, the results show that the proposed scheme can significantly improve the isolation performance of the MIMO antenna array. While the measurement results show minor deviations from simulation, these can be reasonably explained by fabrication and material tolerance. The overall consistency between simulated predictions and empirical results supports the viability and effectiveness of the proposed MSRR-based decoupling structure for practical JCAS applications.

The MSRR decoupling structure proposed in this work features compact size and ease of integration and is suitable for indoor short-range wireless sensing and communication–perception coexistence systems where electromagnetic interference suppression is critical. Although the present prototype operates at 9–10 GHz as a proof of concept, the resonant MSRR unit can be adapted to other frequency bands (including mmWave) through geometry re-optimization and tolerance-aware implementation on appropriate low-loss substrates.

## Data Availability

The datasets generated and analyzed during the current study are available from the corresponding author on reasonable request.
